# The combined risks of reduced or increased function variants in cell death pathway genes differentially influence cervical cancer risk and herpes simplex virus type 2 infection among black Africans and the Mixed Ancestry population of South Africa

**DOI:** 10.1186/s12885-015-1678-y

**Published:** 2015-10-12

**Authors:** Koushik Chattopadhyay, Anna-Lise Williamson, Annapurna Hazra, Collet Dandara

**Affiliations:** 1 Division of Medical Virology and Institute of Infectious Disease and Molecular Medicine (IIDMM), University of Cape Town, Cape Town, Republic of South Africa; 2 National Health Laboratory Service, Groote Schuur Hospital, Cape Town, Republic of South Africa; 3 School of Statistics and Actuarial Sciences, University of KwaZulu-Natal, Durban, Republic of South Africa; 4 Division of Human Genetics, Faculty of Health Science, University of Cape Town, Cape Town, Republic of South Africa; 5 Current address: F. Widjaja Foundation Inflammatory Bowel and Immunobiology Research Institute, Department of Gastroenterology, Cedars-Sinai Medical Center, Los Angeles, CA USA

## Abstract

**Background:**

Cervical cancer is one of the most important cancers worldwide with a high incident and mortality rate and is caused by the human papilloma virus (HPV). Among sexually active women who get infected with human papillomavirus (HPV), a small fraction progresses to cervical cancer disease pointing to possible roles of additional risk factors in development of the disease which include host genetic factors and other infections such as HSV-2. Since cellular apoptosis plays a role in controlling the spread of virus-infections in cells, gene variants altering the function of proteins involved in cell death pathways might be associated with the clearing of virus infections. Activity altering polymorphisms in FasR (−1377G > A and -670A > G), FasL (−844 T > C) and CASP8 (−652 6 N ins/del) genes have been shown to alter the mechanism of apoptosis by modifying the level of expression of their correspondent proteins.

In the present study, we set out to investigate the combined risks of CASP8, FasR, and FasL polymorphisms in cervical cancer, pre-cancerous lesions, HPV infection and HSV-2 infection.

**Methods:**

Participants were 442 South African women of black African and mixed-ancestry origin with invasive cervical cancer and 278 control women matched by age, ethnicity and domicile status. FasR and FasL polymorphisms were genotyped by TaqMan and CASP8 polymorphism by PCR-RFLP. The results were analysed with R using haplo.stats software version 1.5.2.

**Results:**

CASP8 -652 6 N del + FasR-670A was associated with a reduced risk (*P* = 0.019, Combined Polymorphism Score (CPS) = −2.34) and CASP8 -652 6 N ins + FasR-1377G was associated with a marginal increased risk (*P* = 0.047, CPS = 1.99) of cervical cancer among black Africans. When compared within the control group, CASP8 -652 6 N ins + FasR-1377A showed a reduced risk (*P* = 0.023, CPS = −2.28) of HSV-2 infection in both black African and mixed-ancestry population.

**Conclusions:**

Our results show that the combined risks of variants in cell death pathway genes are associated with the cervical cancer as well as the HSV-2 infection in the black African and mixed-ancestry population.

**Electronic supplementary material:**

The online version of this article (doi:10.1186/s12885-015-1678-y) contains supplementary material, which is available to authorized users.

## Background

Cervical cancer is the second most common cancer and the highest cause of cancer related deaths across the globe. Approximately 500, 000 new cases of cervical cancer and nearly 300, 000 deaths (52 %) caused by cervical cancer are reported each year making it the third most common cancer in women of all ages worldwide. Cervical cancer is the second most common cancer in southern Africa with approximately 6,500 new incidents and the second highest cause of cancer mortality with around 3000 cervical cancer related deaths (53 %) annually in women of all ages [1]. Persistent infection with oncogenic types of human papillomavirus (HPV) is the main cause of cervical cancer. Many sexually active women become infected with HPV during their life time but 70–90 % of the individuals positive for HPV DNA are able to clear the infection. Only a small percentage of the women with HPV progress to develop cervical cancer over their time [2]. This suggests a possible role of additional risk factors such as, host and viral genetic factors as well as environmental and lifestyle factors in the development of the cancer of the cervix [3].

Herpes simplex virus type 2 (HSV-2) is a common sexually transmitted virus that causes genital infections. HSV-2 has a high prevalence rate throughout the world, more so in the developing nations. Sub-Saharan Africa particularly harbours a high incidence rate with more women being affected than men [4]. HSV-2 in conjunction with HPV has been implicated as a co-factor for cervical cancer [5]. Once inside a human cell, HSV-2 evades the immune system by overcoming the apoptosis of the cell which is triggered by Fas-FasL-Caspase pathway. Fas mediated cytolysis plays an important role in clearing the HSV-2 from genital epithelium [6].

Apoptosis or programmed cell death is one of the most crucial mechanisms of the human immune system to control viral infection in human body [7]. The interaction between cell surface death receptor Fas (FasR), together with Fas ligand (FasL) activates a caspase-cascade leading to activation-induced cell death (AICD) that eventually leads to apoptosis to eliminate the virus-infected cells [8, 9]. Among various caspases, caspase-8 is central to activating the caspase-cascade [10] and plays one of the most important roles in generating apoptotic signals [9]. Therefore functional polymorphisms in the cell death pathway genes encoding FasR, FasL and caspase-8 might interfere with the apoptotic process associated with the spread and clearance of virus-infections inside human body.

The FasR and FasL genes are mapped on chromosome 10q23 and 1q23 respectively. Three functional single nucleotide polymorphisms (SNPs) in these two genes (FasR-1377G > A, FasR-670A > G and FasL-844 T > C) have been well studied. FasR-1377G > A and FasR-670A > G polymorphisms disrupt the SP1 and STAT1 binding sites respectively leading to reduced Fas gene expression and decreased apoptosis [11, 12]. FasL-844 T > C is observed to show higher basal expression that inhibits the apoptotic activity of the FasR-FasL pathway [13]. These three SNPs have been associated with different diseases [11, 13–24] including cervical cancer in different populations [14, 25–30]. The other important factor in the apoptotic pathway, caspase-8 is encoded by CASP8 gene which is mapped on chromosome 2q33. A six nucleotide deletion of AGTAAG at the position 652 in the promoter region of CASP8 gene (CASP8 -652 6 N ins/del) have been well studied. This six nucleotide deletion destroys the SP1-binding site which reduces the caspase-8 expression and thereby decreasing the apoptotic activity of the caspase-cascade [31]. This polymorphism has been associated with different types of diseases [31–41] including cervical cancer in different populations [31, 41].

No studies to date have attempted to find out the combined risks of all the four polymorphisms from FasR (−1377G > A, −670A > G), FasL (−844 T > C) and CASP8 (−652 6 N ins/del) genes on cervical cancer, HPV infection or HSV-2 infection in any population. We therefore re-analysed our data evaluating the combined risks of CASP8 polymorphism in the presence of both FasR and FasL polymorphisms, on cancer of the cervix susceptibility, HPV infection and HSV-2 infection in South African women of black African and mixed-ancestry origin.

## Methods

### Study participants

Participants in this study were recruited as part of a bigger case–control study to investigate the association of oral contraceptives with cervical cancer [41–43] and included 442 women with invasive cervical cancer (104 black African and 338 women of mixed-ancestry) and 278 control women (52 black African and 226 women of mixed-ancestry) without cancer of the cervix recruited from two previous studies from our lab that investigated the role of FasR (FasR-1377, FasR-670) and FasL (FasL-844) [30] and CASP8 polymorphisms (−652 6 N ins/del) [41] on cervical cancer.

### Herpes simplex virus type 2 (HSV-2) detection, high-risk HPV type detection and papanicolau test

The detection of HSV-2 infection (by HerpeSelect 2 IgG enzyme-linked immunosorbent serologic assay), high-risk HPV infection (by Hybrid Capture II HPV test) and abnormal cytology (by Papanicolaou or Pap smear test) have been described previously [24, 43].

### Clinical specimens, extraction of genomic DNA and genotyping

The collection and storage of clinical specimens, extraction and quantification of genomic DNA and determination of FasR (FasR-1377: rs2234767, assay id, C_12123966_10; FasR-670: rs1800682, assay id, C_9578811_10), FasL (FasL-844: rs763110, custom-designed) and CASP8 (rs3834129:–/AGTAAG;NM_001228.4) polymorphisms have been described previously [24, 41]. All experiments were performed in compliance with relevant laws and institutional guidelines (University of Cape Town Human Research Ethics Committee study approval number, REC REF: 075/2009) and in accordance with the ethical standards of the Declaration of Helsinki. Written informed consent was obtained from each participant of this study.

### Statistical analysis

The genotype distributions for all the polymorphisms were tested for Hardy-Weinberg equilibrium and linkage disequilibrium for cases and controls. Logistic regression was used to test for correlations and associations of different combinations of combined genotypes (CASP8 with FasR/FasL) with cervix cancer status as well as baseline characteristics and secondary outcomes such as age, ethnicity, smoking status, HSV-2 and HIV infection status in cases and controls. HPV and high-risk HPV infection and abnormal cytology status test was done only in the control group (the information for these categories were only available for the controls). Statistical analyses were done with R (the freely available environment for graphics and statistics), using haplo.stats software version 1.5.2 http://CRAN.R-project.org/package=haplo.stats. Broadly, haplo.stats uses the expectation maximisation (EM) algorithm, to calculate maximum likelihood estimates of probabilities of haplotype pairs (one paternal, one maternal) for each subject [44].

## Results

No significant difference in age was observed between cases and controls (*P* = 0.271) (Table 1). 31 % (*n* = 135) cases and 40 % (*n* = 111) controls were tobacco smokers. Smoking was found to be associated with the cases and controls significantly (*P* = 0.01). The HSV-2 status was known for 429 cases and 263 controls. Among cases 68 % (*n* = 291) were positive for HSV-2 infection while among controls 164 (47 black African and 117 women of mixed-ancestry) were positive for HSV-2 infection (62 %). There was no significant difference in the HSV-2 infection between cases and controls. HIV infection status was known for 433 cases and 263 controls. Only 5 % of cases and 4 % of controls were HIV infected which was not found to be significant (Table 1). HPV infection, abnormal cytology and high-risk HPV infection status was known only among controls. HPV infection occurred at a frequency pf 28 % among the 170 controls who had results, while abnormal cytology and high-risk HPV infection was positive in 13 % and 14 %, respectively, among the 269 controls with available data Analysis of only the controls showed a significant association (*P* = 0.013) between abnormal cytology and ethnicity (Table 1).

**Table 1:** Distribution of characteristics of cervical cancer cases and control subjects

Characteristics	Cases		Controls		*P*-value^#^
	Black	Mixed-ancestry	Black	Mixed-ancestry	
Number of subjects	104	338	52	226	
Age (years), mean ± SD	43.7 ± 9.3	45.9 ± 8.1	40.8 ± 8.9	45.5 ± 8.1	0.271
Smoking					
Positive	82	53	44	67	0.012
Negative	22	285	8	159	
HSV-2 infection					
Infected	93	198	47	117	0.161
Non-infected	10	128	3	96	
HIV infection					
Infected	13	9	4	7	0.589
Non-infected	89	322	46	206	
HPV infection					
Infected			12	35	0.539
Non-infected			26	97	
Abnormal cytology					
Positive			12	23	0.013
Negative			39	195	
High-risk HPV infection					
Positive			7	32	0.862
Negative			44	186	

^#^*P*-values were based on chi square test and for black and mixed-ancestry combined

The observed genotype frequencies for all the polymorphisms were found to be in Hardy-Weinberg equilibrium (HWE) except for CASP8 -652 6 N ins/del which was slightly out of HWE (*P* = 0.045) in mixed-ancestry controls [30, 41]. FasR polymorphisms (−1377 and −670) were in strong linkage disequilibrium (LD) (*P* < 0.05) except in black African controls (*P* = 0.078) [30]. We have previously reported that the allele and genotype frequency distributions for FasR/FasL differ significantly between the ethnic groups (black African and mixed-ancestry) investigated [30]. Among all the baseline characteristics and secondary outcomes only smoking was found to be a significant confounding factor for cancer of the cervix as mentioned above. Therefore, all analyses were subsequently adjusted for both ethnicity and smoking by including in logistic regression models as covariates.

CASP8 polymorphism (CASP8 -652 6 N ins/del) was combined with any (and all) of the FasR (−1377, −670) and FasL (−844) polymorphisms which gave rise to 7 combinations of polymorphisms (total 52 different combinations) as shown in Additional file 1. None of the combinations showed any significant association (*P* < 0.05) with cancer of the cervix (both population combined) (Table 2). When stratified for different populations, the mixed-ancestry population again did not show any significant association (*P* < 0.05) with the disease (Table 2). However, the black Africans showed a marginal increased risk with CASP8 -652 6 N ins + FasR-1377G (*P* = 0.047, Combined Polymorphism Score (CPS) = 1.99) and cervical cancer and a reduced risk with CASP8 -652 6 N del + FasR-670A (*P* = 0.019, CPS = −2.34) with the same disease (CPS is similar as a haplotype score; a positive value suggests a positive association and a negative value suggests a negative association with the disease under investigation) (Table 2). The combined risks of CASP8 -652 6 N del + FasR-670A polymorphism showed a reduced risk of cervical cancer even when combined with FasR-1377 and FasL-844 polymorphisms (Table 2).

**Table 2:** Association statistics for combined polymorphisms of CASP8 (− 652 6 N ins/del), FasR-1377 (G > A), FasR-670 (A > G) and FasL-844 (T > C) for cervical cancer cases and controls

Black Africans and Mixed Ancestry combined			
Haplotype or Genotype combinations	Cases, *n* = 442 (frequency)	Controls, *n* = 278 (frequency)	*P*-value (global)
* CASP8 + FasR-1377*	87 (49)	92 (51)	0.531
* CASP8 + FasR-670*	98 (50)	99 (50)	0.63
* CASP8 + FasL-844*	97 (50)	98 (50)	0.738
* CASP8 + FasR-1377 + FasR-670*	86 (48)	92 (52)	0.813
* CASP8 + FasR-1377 + FasL-844*	85 (48)	91 (52)	0.771
* CASP8 + FasR-670 + FasL-844*	95 (50)	97 (50)	0.937
* CASP8 + FasR-1377 + FasR-670 + FasL-844*	84 (48)	90 (52)	0.978
Among Black Africans			
Combined Polymorphisms	Cases, n = 104 (frequency)	Controls, *n* = 52 (frequency)	*P*-value (global)
* CASP8 + FasR-1377*	96 (66)	49 (34)	0.138 (global P)
−652 6 N ins and G	(41)	(28)	0.047 (CPS = 1.99)
* CASP8 + FasR-670*	103 (67)	51 (33)	0.028 (global P)
−652 6 N del and A	(6)	(19)	0.019 (CPS = −2.34)
* CASP8 + FasL-844*	103 (67)	51 (33)	0.1 (global P)
* CASP8 + FasR-1377 + FasR-670*	96 (67)	48 (33)	0.131 (global P)
−652 6 N del, G and A	(6)	(19)	0.019 (CPS = −2.35)
* CASP8 + FasR-1377 + FasL-844*	95 (66)	48 (34)	0.181 (global P)
* CASP8 + FasR-670 + FasL-844*	102 (67)	50 (33)	0.047 (global P)
−652 6 N del, A and C	(1)	(7)	0.018 (CPS = −2.36)
* CASP8 + FasR-1377 + FasR-670 + FasL-844*	95 (67)	47 (33)	0.115 (global P)
−652 6 N del, G, A and C	(9)	(7)	0.027 (CPS = −2.21)
Among Mixed-Ancestry group			
Haplotypes	Cases, n = 338 (frequency)	Controls, n = 226 (frequency)	*P*-value (global)
* CASP8 + FasR-1377*	289 (58)	208 (42)	0.747
* CASP8 + FasR-670*	330 (59)	225 (41)	0.966
* CASP8 + FasL-844*	326 (59)	222 (41)	0.909
* CASP8 + FasR-1377 + FasR-670*	282 (58)	207 (42)	0.971
* CASP8 + FasR-1377 + FasL-844*	280 (58)	204 (42)	0.924
* CASP8 + FasR-670 + FasL-844*	320 (59)	221 (41)	0.991
* CASP8 + FasR-1377 + FasR-670 + FasL-844*	275 (58)	203 (42)	0.996

*P*-values and Combined Polymorphism Scores (CPSs) are for test of combined polymorphism association with cervix cancer risk, adjusted for smoking and ethnicity for total cases and controls and smoking only for stratified population groups. CPS = combined polymorphism score. Cases = Women with cancer of the cervix, Controls = Women without cancer of the cervix

Comparing the different polymorphisms in smoking positive and HSV-2 positive cases and controls did not show any significant association (results not shown here) either.

The combined risks of the polymorphisms were also investigated only within the control group, as the controls represent the larger healthy South-African female population, unlike the cervical cancer group that is affected by a disease. When HSV-2 infection status was compared in the control group for the different combinations of the polymorphisms, a reduced risk was found with CASP8 -652 6 N ins + FasR-1377A (*P* = 0.023, CPS = −2.28) and HSV-2 infection (Table 3). The combined risks of these two polymorphisms showed a reduced risk of HSV-2 infection even when combined with FasR-670 and FasL-844 polymorphisms (Table 3). Comparing the other parameters such as, smoking, high-risk HPV infection and abnormal cytology status in the control group did not yield any significant association with any of the parameters mentioned (results not shown here).

**Table 3:** Association statistics for combined polymorphisms of CASP8 (− 652 6 N ins/del), FasR-1377 (G > A), FasR-670 (A > G) and FasL-844 (T > C) for HSV-2 infection status only in controls

Combined Polymorphisms	HSV-2 infected Frequency (*n* = 164)	HSV-2 non-infected Frequency (*n* = 99)	Combined Polymorphism Score	*P*-value
*Casp8 + FasR-1377* (global *P* = 0.134)				
−652 6 N ins and A	7	12	−2.28	0.023
*Casp8 + FasR-1377 + FasR-670* (global *P* = 0.164)				
−652 6 N ins, A and G	7	12	−2.17	0.03
*Casp8 + FasR-1377 + FasR-670 + FasL-844* (global *P* = 0.324)				
−652 6 N ins, A, G and T	16	22	−2.13	0.033

*P*-values and Combined Polymorphism Scores (CPSs) are for test of combined polymorphism association with HSV-2 infection in controls, adjusted for ethnicity and smoking. P-values next to combined polymorphism names are for joint model, others are for CPSs of specific combined polymorphism compared to a reference combined polymorphism (not shown here)

## Discussion

The cell death pathway is one of the most essential pathways in human immune system. This pathway is central to and regulates cellular apoptosis which is one of the mechanisms by which the immune system controls infections caused by pathogenic viruses [7]. Disruptions in cell death pathway can lead to impaired apoptotic mechanism which may affect the ability of the immune system to control the spread of virus-infections in cells. Three functional polymorphisms in FasR (FasR-1377, FasR-670) and FasL genes (FasL-844) and one in CASP8 gene (CASP8 -652 6 N ins/del), all in cell death pathway are known to disrupt the apoptotic mechanism by altering the level of expression of Fas and caspase-8 proteins, respectively [11–13, 31]. All the four polymorphisms have been investigated in different types of cancers such as breast [32, 33, 37], bladder [19, 40], lung [20, 23], squamous cell carcinoma [15, 22], colorectal [35], pancreatic [36], different leukaemia [11, 16], cervical [14, 25–31, 41] and several other pathological conditions [11, 13–23, 31–41], all presenting with conflicting results. Previously we have investigated these four polymorphisms in cervical cancer in South African women of black African and mixed-ancestry origin [24, 30, 41]. Comparing women with invasive cervical cancer with age, ethnicity and domicile (urban/rural) matched controls did not show any significant association for FasR and FasL polymorphisms with cervical cancer [30]. However, a secondary analysis only in the control group found a statistically significant association with FasR-1377A (*P* = 0.008) and FasR-1377/FasR-670 AG haplotype (*P* = 0.0001) with reduced risk of HSV-2 infection [24]. When CASP8 -652 6 N ins/del polymorphism was compared between cervical cancer cases and controls no significant association with cervical cancer was observed [41]. However, further analysis within only controls showed an association (positive) of CASP8 -652 6 N del/del (P = 0.03) polymorphism with high-risk HPV infection (only among black Africans) and a weak association (positive) with abnormal cytology (*P* = 0.048) suggesting a susceptible role for this genotype in the development of pre-cancers but not in cervical cancer [41]. The combined risks of all the four polymorphisms of FasR, FasL and CASP8 on cervical cancer, HPV infection, pre-cancerous lesions and HSV-2 infection have not been investigated in any population yet.

Since all the four polymorphisms play vital roles in the cell death pathway leading to altered mechanism of cellular apoptosis it is valuable to investigate the combined risks of CASP8 polymorphism with any (and all) of the FasR/FasL polymorphisms in cervical cancer, pre-cancerous lesions, HPV infection and HSV-2 infection. None of the combinations of the polymorphisms had any significant association with cancer of the cervix in both populations combined and also when stratified for mixed-ancestry population, which is in accordance with our previous individual results with FasR/FasL [30] and CASP8 polymorphisms [41]. However, when stratified among only black Africans a new reduced risk for cervical cancer was observed with CASP8 -652 6 N del combined with FasR-670 A allele (*P* = 0.019, CPS = −2.34). In the same population, a marginal susceptible risk to cervical cancer was also observed when CASP8 -652 6 N ins was combined with FasR-1377 G allele (*P* = 0.047, CPS = 1.99). This could be due to different factors. The black Africans are a homogenous population with well defined genetic conformation unlike the mixed-ancestry population that carries a highly mixed genetic makeup. It is also well known that disease susceptibility varies from population to population due to varying level of genetic markers present in various population.

When the combined risks of the polymorphisms were investigated only in the controls, a reduced risk for HSV-2 infection was observed with CASP8 -652 6 N ins + FasR-1377A. This is in consistence with our previous results [24] that showed a reduced risk for HSV-2 infection with FasR-1377A allele alone in the control group as mentioned above. CASP8 promoter polymorphism individually have also been associated with reduced risk of cutaneous melanoma, lung, esophageal, gastric, breast, colorectal and cervical cancer [31, 34] in different population even though in our own population we found an increased risk of this polymorphism with cervical cancer [41].

None of the combinations of the polymorphisms had any significant association with any other parameters in the control group suggesting the combined risks of the polymorphisms are not associated with smoking, high-risk HPV infection and abnormal cytology status in the larger South-African female population.

## Conclusions

The present study showed a reduced risk to cervical cancer combining CASP8 -652 6 N del with FasR-670 A allele (*P* = 0.019, CPS = −2.34) and a marginal susceptible risk to cervical cancer with CASP8 -652 6 N ins + FasR-1377G (*P* = 0.047, CPS = 1.99), only in black Africans. This study also showed a reduced risk to HSV-2 infection with CASP8 -652 6 N ins + FasR-1377A in the control group. The results must be interpreted cautiously. This was a sub-group analysis of our original study and the results could be influenced due to several possible population or biological confounding factors that are hitherto unrecognized. Future studies on larger cohorts are warranted to further confirm these findings and to elucidate the mechanisms involved.

## Additional file

Additional file 1: **The Genotype combinations observed when analysing CASP8 (- 652 6 N ins/del),** **FasR-1377** **(G > A),** **FasR-670** **(A > G) and** **FasL-844** **(T > C)** **genetic variants.** (DOC 42 kb)
