# Supplementary material for: The combined risks of reduced or increased function variants in cell death pathway genes differentially influence cervical cancer risk and herpes simplex virus type 2 infection among black Africans and the Mixed Ancestry population of South Africa
Source: BMC Cancer. 2015 Oct 12;15:680. doi: 10.1186/s12885-015-1678-y (PMC4603903; doi:10.1186/s12885-015-1678-y)
Supplement: Additional file 1: — The Genotype combinations observed when analysing CASP8 (- 652 6 N ins/del), FasR-1377 (G > A), FasR-670 (A > G) and FasL-844 (T > C) genetic variants. (DOC 42 kb) [file 12885_2015_1678_MOESM1_ESM.doc]

# Additional files

### Additional file 1 – The Genotype combinations observed when analysing CASP8 (- 652 6N ins/del), FasR-1377 (G>A), FasR-670 (A>G) and FasL-844 (T>C) genetic variants

| **Combinations of polymorphisms** | **Cases**  **number of cases (frequency)** | **Controls**  **number of controls (frequency)** | **P-value^#^** |
| --- | --- | --- | --- |
| 1. ***CASP8+FasR-1377*** 2. CASP8 -652 6N ins+FasR-1377G 3. CASP8 -652 6N ins+FasR-1377A 4. CASP8 -652 6N del+FasR-1377G 5. CASP8 -652 6N del+FasR-1377A | 87 (49)  (35.50)  (9.22)  (35.24)  (7.05) | 92 (51)  (36.62)  (8.83)  (39.84)  (6.62) | 0.53  0.72  0.45  0.30  0.65 |
| 1. ***CASP8+FasR-670*** 2. CASP8 -652 6N ins+FasR-670A 3. CASP8 -652 6N ins+FasR-670G 4. CASP8 -652 6N del+FasR-670A 5. CASP8 -652 6N del+FasR-670G | 98 (50)  (17.64)  (33.32)  (13.72)  (34.3) | 99 (50)  (17.82)  (31.68)  (16.83)  (32.67) | 0.63  0.79  0.30  0.13  0.81 |
| 1. ***CASP8+FasL-844*** 2. CASP8 -652 6N ins+FasL-844T 3. CASP8 -652 6N ins+FasL-844C 4. CASP8 -652 6N del+FasL-844T 5. CASP8 -652 6N del+FasL-844C | 97 (50)  (33.95)  (15.52)  (33.95)  (13.58) | 98 (50)  (31.36)  (16.66)  (34.3)  (14.7) | 0.74  0.26  0.68  0.88  0.33 |
| 1. ***CASP8+FasR-1377+FasR-670*** 2. CASP8 -652 6N ins+FasR-1377G+FasR-670A 3. CASP8 -652 6N ins+FasR-1377G+FasR-670G 4. CASP8 -652 6N ins+FasR-1377A+FasR-670G 5. CASP8 -652 6N del+FasR-1377G+FasR-670A 6. CASP8 -652 6N del+FasR-1377G+FasR-670G 7. CASP8 -652 6Ndel+FasR-1377A+FasR-670G | 86 (48)  (15.48)  (20.64)  (8.6)  (12.04)  (23.22)  (6.79) | 92 (52)  (16.56)  (20.24)  (8.74)  (15.64)  (23.92)  (6.72) | 0.81  0.78  0.44  0.54  0.13  0.95  0.75 |
| 1. ***CASP8+FasR-1377+FasL-844*** 2. CASP8 -652 6N ins+FasR-1377G+FasL-844T 3. CASP8 -652 6N ins+FasR-1377G+FasL-844C 4. CASP8 -652 6N ins+FasR-1377A+FasL-844T 5. CASP8 -652 6N ins+FasR-1377A+FasL-844C 6. CASP8 -652 6N del+FasR-1377G+FasL-844T 7. CASP8 -652 6N del+FasR-1377G+FasL-844C 8. CASP8 -652 6N del+FasR-1377A+FasL-844T 9. CASP8 -652 6N del+FasR-1377A+FasL-844C | 85 (48)  (10.92)  (16.94)  (4.92)  (0.000004)  (36.44)  (7.11)  (7.21)  (1.45) | 91 (52)  (24.37)  (13.12)  (76.80)  (2.20)  (26.46)  (12.24)  (3.77)  (22.07) | 0.77  0.07  0.92  0.34  0.43  0.06  0.85  0.13  0.80 |
| 1. ***CASP8+FasR-670+FasL-844*** 2. CASP8 -652 6N ins+FasR-670A+FasL-844T 3. CASP8 -652 6N ins+FasR-670A+FasL-844C 4. CASP8 -652 6N ins+FasR-670G+FasL-844T 5. CASP8 -652 6N ins+FasR-670G+FasL-844C 6. CASP8 -652 6N del+FasR-670A+FasL-844T 7. CASP8 -652 6N del+FasR-670A+FasL-844C 8. CASP8 -652 6N del+FasR-670G+FasL-844T 9. CASP8 -652 6N del+FasR-670G+FasL-844C | 95 (50)  (12.64)  (4.47)  (20.81)  (10.93)  (6.56)  (6.46)  (26.51)  (6.65) | 97 (50)  (12.03)  (4.85)  (19.98)  (11.16)  (8.05)  (8.63)  (25.61)  (6.69) | 0.94  0.82  0.60  0.34  0.91  0.35  0.11  0.61  0.87 |
| 1. ***CASP8+FasR-1377+FasR-670+FasL-844*** 2. CASP8 -652 6N ins+FasR-1377G+FasR-670A+FasL-844T 3. CASP8 -652 6N ins+FasR-1377G+FasR-670A+FasL-844C 4. CASP8 -652 6N ins+FasR-1377G+FasR-670G+FasL-844T 5. CASP8 -652 6N ins+FasR-1377G+FasR-670G+FasL-844C 6. CASP8 -652 6N ins+FasR-1377A+FasR-670G+FasL-844T 7. CASP8 -652 6N ins+FasR-1377A+FasR-670G+FasL-844C 8. CASP8 -652 6N del+FasR-1377G+FasR-670A+FasL-844T 9. CASP8 -652 6N del+FasR-1377G+FasR-670A+FasL-844C 10. CASP8 -652 6N del+FasR-1377G+FasR-670G+FasL-844T 11. CASP8 -652 6N del+FasR-1377G+FasR-670G+FasL-844C 12. CASP8 -652 6N del+FasR-1377A+FasR-670G+FasL-844T | 84 (48)  (10.92)  (3.95)  (12.6)  (6.55)  (5.46)  (3.02)  (5.80)  (5.71)  (17.64)  (4.62)  (5.46) | 90 (52)  (10.8)  (4.59)  (12.6)  (7.83)  (6.03)  (2.34)  (7.56)  (7.92)  (18.9)  (4.68)  (5.04) | 0.98  0.82  0.58  0.39  0.88  0.68  0.59  0.36  0.11  0.75  0.86  0.66 |

^#^All P-values are un-adjusted.
